# Reproducibility of digital pathology features extracted from deep learning and foundational AI models on sequential tissue slides

**DOI:** 10.1038/s41598-025-30947-w

**Published:** 2025-12-06

**Authors:** Jagadheshwar Balan, Nicholas B. Larson

**Affiliations:** Quantitative Health Sciences, Mayo Clinic, Rochester, Minnesota USA

**Keywords:** Digital pathology, Nuclei segmentation, Cell type features, Spatial features, Foundation model embeddings, Computational biology and bioinformatics, Medical research

## Abstract

Numerous deep learning (DL) and foundational models (FMs) designed for digital pathology analysis employ advanced feature extraction techniques from digitized hematoxylin and eosin (H&E) slides obtained from tissue sections. These extracted features may serve as the input for complex models enabling automated classification tasks, slide searching, and other various medical inferences. Subtle variations in morphology observed in distinct cross sections of the same tissue sample may introduce variability that may be of little to no clinical relevance. The reproducibility of features derived from DL models and FMs on ostensibly identical tissue cross sections remains largely unexplored. In this study, we aimed to characterize the reproducibility of features extracted from three sequential cross sections of 50 independent normal prostate samples. Slides were sectioned at approximately 50 μm intervals and we extracted cell-type specific counts, proportions, and spatial features per slide using DL models, along with feature embeddings from popular digital pathology FMs. Reproducibility of the features across sequential cross sections of tissues was assessed using intra-class correlation coefficient (ICC) for DL model features, while extracted FM embedding distributions were evaluated via maximum mean discrepancy (MMD), and Wasserstein’s distance. The median [IQR] ICC estimates for cell-type specific proportion and spatial features from the cross sections was 0.838 [0.764, 0.897], and 0.873 [0.809, 0.923] respectively. The bootstrapped mean differences of ICC estimates for the features were significantly lower (p-value < 0.05) for distant cross sections compared to the two neighboring cross sections. The feature embeddings derived from state-of-the-art digital pathology FMs showed an overall high agreement with the median [IQR] MMD of 0.06 [0.016, 0.169], and median [IQR] Wasserstein’s distance of 0.021 [0.011, 0.038]. These findings demonstrate that the features derived from multiple cross sections of the same tissue exhibit overall high reproducibility, while also illustrating the expected increase in intra-sample variability as a function of physical distance between sections.

**Supplementary Information:**

The online version contains supplementary material available at 10.1038/s41598-025-30947-w.

## Introduction

Digital pathology implements novel imaging systems to capture high-resolution whole slide images (WSIs) of tissues and uses advanced image processing techniques to support a wide range of translational and clinical applications, including cell segmentation and classification, tumor grading, cancer detection, prognostic modeling, image retrieval, and the integration of multi-omics data^1–4^. The availability of large WSI datasets and associated curated annotations has resulted in the development of advanced models that have demonstrated clinical potential across a wide variety of tasks^1–4^. Additionally, regulatory approvals of digital pathology instruments and associated software have bolstered scientific advancements in the field, with models being developed and used for user-prompt based approaches aiming to answer medically relevant questions^1,5,6^.

The advanced computational approaches for WSI analyses use complex model architectures that incorporate deep neural networks and vision transformers^3,7,8^. These model architectures are trained using morphological features, topological features, and complex feature embeddings and have facilitated automating several clinical tasks that are otherwise resource intensive^1,9,10^. The features extracted from the WSIs serve as the inputs of the artificial intelligence (AI) models to predict and classify across a wide range of tasks^2,3,6,9–13^. It is important to note that the WSIs represent a single cross section of tissue, which may introduce sampling bias^14^. Changes in morphology and topology have been observed in distinct cross sections of the same tissue sample^15^; therefore, it is essential to evaluate the consistency of features within these sections. For example, many translational studies model bulk-RNA gene expression profiles digital pathology features as predictors or covariates^7,16–18^, and feature instability across tissue cross sections might result in ambiguous mechanistic links between morphology and gene expression. As AI-assisted digital pathology expands in scope and accessibility, evaluating feature reproducibility across tissue cross sections is essential. This process has the potential to enhance reliability, particularly when robust, reproducible features are utilized for model training and inference. Additionally, clinical decisions frequently depend on threshold values derived from individual measurements. If the features used by the AI-based computational pathology vary across different cross sections, this intra-sample variability could raise concerns regarding their reliability and suitability for clinical application. However, the reproducibility of features extracted based on the complex AI models from sequential cross sections of the same tissue sample remains largely unexplored and there is no consensus on acceptance criteria for intra-sample variability of features within tissue cross sections. Reproducible features reduce misclassification at therapeutic cutoffs, prevent inappropriate exclusion in trials, and potentially support stable longitudinal assessments such as immunotherapy response. When upstream features are stable and bias-controlled, downstream digital pathology AI models exhibit better calibration and reproducibility.

The relative abundance of specific types of cells in various biological contexts, such as cancer, can provide crucial insights into immune response, gene expression, and patient survival^19^. Additionally, spatial localization of specific cell types within the tissue and adjacency to other cell types can influence gene expression, fundamentally shaping cell identity and function within complex tissues^20^. In this study, we evaluate the reproducibility of various digital pathology features of sequential cross sections derived from 50 normal prostate tissue samples. We extract features using popular segmentation and foundational models using digitized pathological hematoxylin and eosin (H&E) slides from sequential cross sections of normal prostate tissue samples. Features evaluated include cell type proportions, and spatial features of the diverse types of nuclei present in the WSIs derived using deep learning (DL) models. Finaly, we also evaluate reproducibility of feature embeddings derived from popular vision transformer-based FMs.

**Fig. 1:**
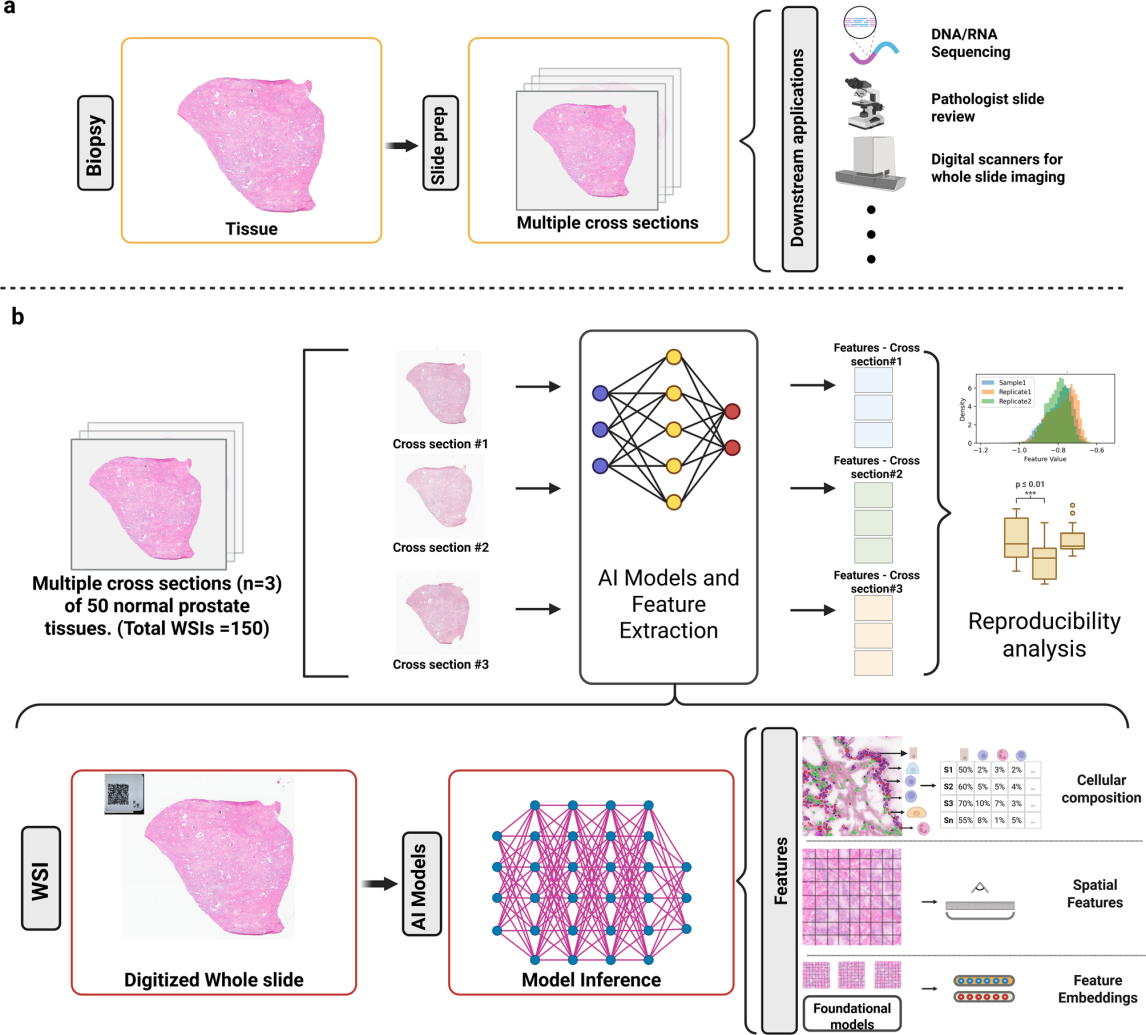
(**a**) Illustration of standard practice of producing multiple cross sections from a tissue for various downstream applications that include sequencing, slide review and digital pathology. (**b**) Workflow of our study that processes sequential cross sections through same AI models to extract features and compare the features across the cross sections to assess reproducibility. (Created in BioRender. Balan, J. (2025) https://BioRender.com/oznmra2).

## Methods

### Sample cohort and slide digitization

An overview of our study design, the dataset, and methods to assess feature reproducibility across tissue cross sections are detailed in Fig. 1. Our study leveraged data generated from a previous study of normal prostate tissue, which was acquired from an archive collection of fresh frozen material obtained from patients with radical prostatectomy or cystoprostatectomy^21^. The previous study applied a sectioning protocol to collect multiple samples for DNA and RNA extraction, with flanking sections cut for H&E slide preparation to monitor for consistency in tissue quality (Supplementary Fig. 1). Briefly, fresh frozen tissue specimens were first sectioned at approximately 5 μm for an initial slide, followed by approximately 50 μm of tissue that was sectioned and placed into a tube for RNA extraction. A second H&E slide section was then cut and prepared to prior to an additional 50 μm of tissue for a second RNA tube. Finally, a third H&E section was cut for slide preparation prior to a similar process continuing for tissue sectioning for DNA extraction. Larger sections were cut for two additional tubes for DNA extraction (each approximately 250 μm), ultimately yielding five total H&E slides. All the slides were stained and prepared for the study in a single batch to mitigate batch effects using a standard operating procedure (see Supplementary Text).

To assess reproducibility of features across sequential cross sections, the first three H&E slides that flanked sections collected for RNA were selected. Slides were digitized with the same settings using the Aperio Leica GT-450 digital pathology scanner, with a resolution of 40X magnification and 0.263 microns per pixel. Manual quality control checks included careful review to rule out cracks, bubbles, and any physical damage to the tissue slides prior to scanning to ensure high quality WSIs. Among the three sequential cross sections per tissue digitized in the study (designated C1, C2, and C3), C1 vs. C3 is referred to as distant with an approximate inter-slide distance of 105 μm, compared to neighboring closer sections C1 vs. C2 and C2 vs. C3 at inter-slide distances of approximately 50 μm.

### Feature extraction using AI models

#### Cell and nuclei segmentation features

The nuclei segmentation and cell type identification task was performed using TIAToolBox, a suite of libraries and tissue image analytics pipelines^3,8,22,23^. Briefly, HoVer-Net model trained on PanNuke and HoVer-Net model trained on MoNuSac were used for nuclei segmentation and cell type assignments. The segmented nuclei and assigned cell types from the two models were integrated using cellmerge^24^ to ensure inclusion of a diverse set of cell types. A total of eight cell types were included in the final merged results that included epithelial cells (neoplastic, non-neoplastic and uncategorized), inflammatory cells (that overlapped between HoVer-Net PanNuke and HoVer-Net MoNuSac), lymphocytes, neutrophils, connective cells, and dead cells. Four Tesla V100 Graphical Processing Unity (GPU) machines were used in parallel to accelerate inference times. Final set of cell and nuclei features (*n* = 16) included the number of nuclei by each cell type and proportion of each cell type present in the WSI (Supplementary Table 1).

#### Spatial features

The merged nuclei segmentations provided centroids (x-y coordinates for each nucleus on the WSI) and associated cell types, which were then utilized to extract spatial features. The spatial features extracted from the WSIs were categorized into three major categories (Supplementary Table 1). The first category quantifies the area and size of the nuclei segmentations by the cell type. The second category effectively quantifies the number of times a specific cell type occurs to another by computing pairwise counts of closest neighbor of a cell type to another. The third category quantifies the presence of diverse cell types for each cell type by calculating Shannon diversity index. To simplify and reduce the spatial feature space while still capturing the cell diversity, similar sub-cell types that were sparse were grouped according to their larger cell types for the spatial features (Supplementary Table 2). Under the first category of features (*n* = 15), the coefficient of variation of the Voronoi polygon area for each cell type along with the mean and standard deviation of edge lengths based on Delaunay triangulation of nuclei by each cell type was computed^25–27^. Voronoi polygons were generated using scipy python module (version 1.14.1, this version is used throughout the study)^28^. The Voronoi polygons are prone to edge effects, where the outer polygons generated around the periphery of tissue area are not bound by defined edges (Supplementary Fig. 2). The edge effects were handled by applying an outlier detection technique, where polygon areas outside of 1.5 interquartile range (IQR) away from first and third quartiles were excluded as per Tukey’s inner fence rule for removing outliers^29^. In the second category of spatial features (*n* = 25), approximate nearest neighbors algorithm (Annoy)^30^ was used to identify the nearest neighboring nuclei for each segmented nuclei and a matrix of pairwise count of nearest neighbor for each cell type was computed. This feature set yielded a count matrix, quantifying the presence of other neighboring cell types for each cell type. In the third category of spatial features (*n* = 5), a Euclidean distance of 100 was used to identify closest neighbors for each nuclei using the Annoy algorithm, and a mean Shannon diversity index for each cell type was calculated. To select the Euclidean distance threshold, a WSI was randomly selected from our cohort, various thresholds were derived to identify at least 5, 10, and 15 neighboring cells. The corresponding distance thresholds were 53.2 units for 5 neighbors, 81.3 units for 10 neighbors, and 100.4 units for 15 neighbors. To avoid including cells that are unlikely to influence the spatial arrangement of the cell in question, we limited our analysis to maximum of 15 neighbors. Based on the empirical choice of 15 neighbors, a distance threshold of 100 units was adopted to encompass at least 15 neighboring cells around each cell, and the Shannon diversity index feature was calculated. This feature quantifies the abundance or lack of diversity of other neighboring cell types for each cell type.

#### Features from foundational models

TIAToolbox^8^ was used to extract feature embeddings from popular pathology foundation models including UNI, Prov-GigaPath, and H-optimus-0^9–11^. The popular models were chosen based on their performance in a recent study that highlights the need for robust foundation models for digital pathology^31^. Briefly, an Otsu threshold was applied to remove the background, and features were extracted at a whole slide level where each feature vector represents the embeddings extracted from the pre-trained vision encoder trained by the individual models. All the FMs evaluated in our study use a transformer architecture based on DINOV2 model^32^. The output from each FM is a 1024-dimensional vector of embeddings extracted by the vision encoder for a $\:224\:\times\:224$ tile in the WSI. Hence, the number of features from the foundational models are a $\:m\:\times\:1024$ dimensional vector, where $\:m$ is the total number of $\:224\:\times\:224$ tiles in the WSI.

#### Statistical analysis to assess feature reproducibility

To compare the cell type and spatial features across the cross sections, intra-class correlation (ICC) estimates and corresponding 95% confidence intervals (CIs) were calculated in a pairwise-fashion between each cross section of the tissue sample using pingouin python module (version 0.5.5). The implementation of ICC by the pingouin module uses the ANOVA estimator approach^33^. ICC estimates traditionally range from 0 to 1, and higher values indicate an increased agreement of features between the cross sections. Interpretation of ICC estimates was based on Cicchetti et al.^34^, such that estimates < 0.40 were considered poor, 0.40–0.59 as fair, 0.60–0.74 as good, and 0.75-1.00 as excellent. ICC estimates were calculated at a feature level by unique pair-wise cross section comparisons, while accounting for distant versus closer cross sections (C1 vs. C3, C1 vs. C2, and C2 vs. C3). To assess if the differences in ICC values between distant versus closer cross sections were statistically significant, cluster bootstrapping was used. Cluster bootstrapping was performed for grouping the structure of the data by resampling within each comparison group C1 vs. C3, C1 vs. C2, and C2 vs. C3. A total of 10,000 bootstrap samples were performed and in each iteration, observations were resampled with replacement within each comparison group while preserving the grouping structure. For each resample, mean ICC value per group was calculated and this resulted in bootstrapped distribution of mean ICC value per group. Pairwise differences between the group means were computed and two-tailed p-values were calculated to assess statistical significance.

In our study, we evaluated metric comparisons at the full feature size of $\:m\:\times\:1024$ vector, where $\:m$ is the total number of $\:224\:\times\:224$ tiles in the WSI. To compare the feature embeddings extracted from the FMs, including UNI, Prov-GigaPath, and H-optimus-0, the embeddings from each sequential cross section ($\:m\:\times\:1024$ vector, where $\:m$ is the total number of $\:224\:\times\:224$ tiles in the WSI) were reduced to $\:m\:\times\:\:$100 dimensions using UMAP^35^. Several dimensions for UMAP (i.e., 100, 250, 500 and 750) were evaluated on the WSI tiles, and 100 was chosen because neighborhood fidelity (trustworthiness), global pairwise distance monotonicity (based on non-identical tile pairs), and inter-slide dissimilarity (using Maximum Mean Discrepancy with an RBF kernel) plateaued at 100 dimensions, providing substantial compute time savings for downstream metrics. A pairwise comparison of features from each cross section on a tissue was performed by computing Maximum Mean Discrepancy (MMD)^36^, and Wasserstein’s distance^37^ on the 100 UMAP-derived dimensions. While MMD and Wasserstein distance have been used previously in comparing distributions of real data versus generated data for evaluating how closely a generative model approximates real data distribution^38^, there are no universal reference ranges for these metrics. To assess if the sequential cross sections result in similar features from the FMs, we compared the MMD and Wasserstein distance metrics between sample embeddings of same sample’s tissue and unrelated sample pairs from our cohort. A $\:150\:X\:150$ dis/similarity distance matrix was generated for each model evaluated and median of distance metrics for each model were compared between related and dissimilar samples (self-pairs were excluded from this analysis). To assess statistical significance, a permutation test was performed for each model and metric (MMD and Wasserstein distance). The permutation test was performed to assess if the within-group values (related samples) were significantly smaller than between-group values (unrelated samples). A total of 10, 000 permutations were performed with the alternative hypothesis that the within-group values are smaller than the between-group values. p-values were obtained from the empirical null distribution generated by label shuffling, with a fixed random seed for reproducibility. p-values < 0.05 would indicate that unrelated samples and related samples are significantly more dissimilar, and the metrics of related samples are significantly lower than unrelated samples. Finally, to assess if the FMs are confounded by presence of any stain differences, we perform stain normalization on all the WSIs using Vahadane stain normalization method and share the results based on this sensitivity analysis. If the results are deviant from the raw slides, then the FM embedding differences may be attributable to the stain differences.

## Results

### Reproducibility of nuclei segmentation and spatial features

**Fig. 2:**
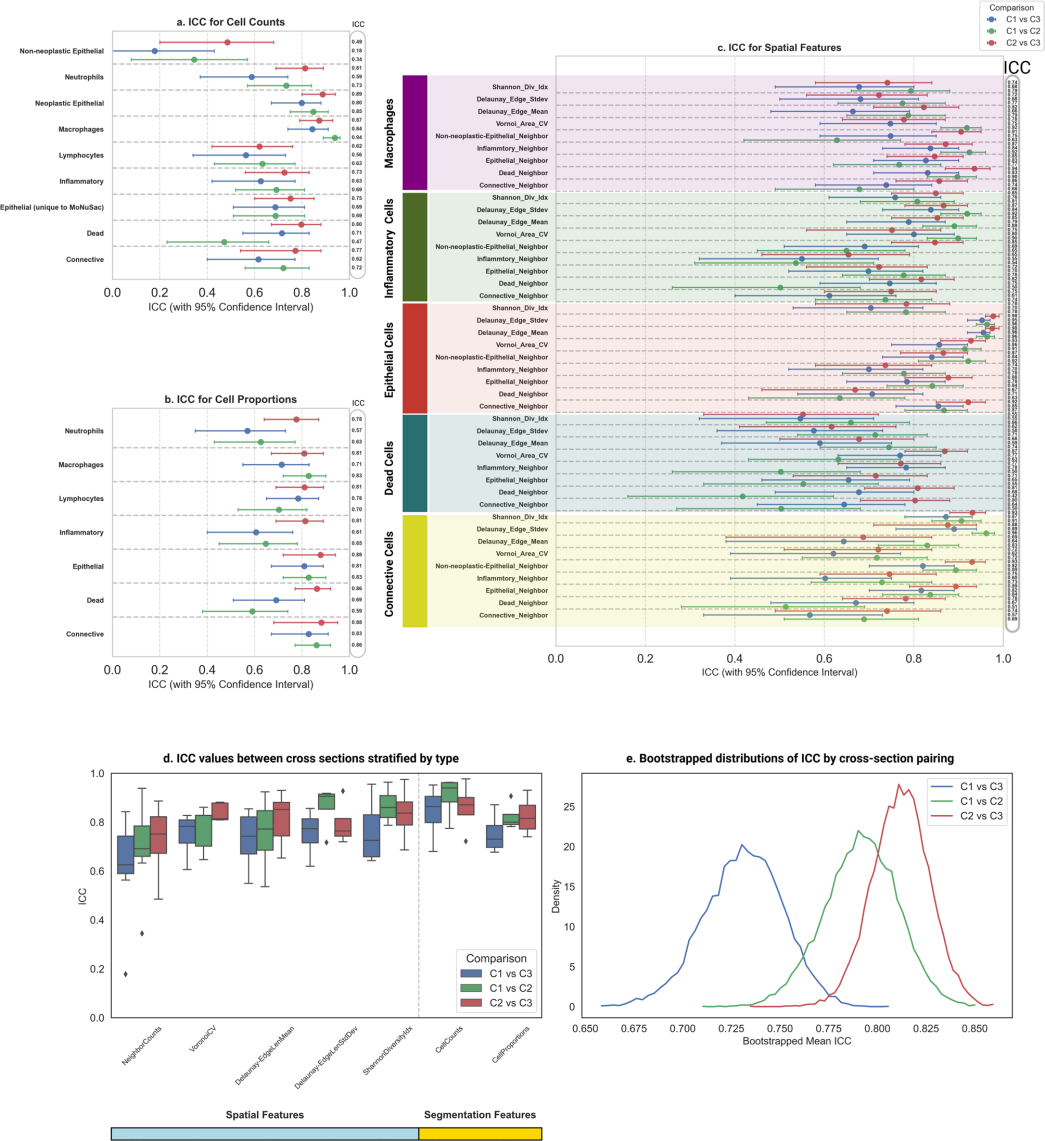
(**a**) Forest plot showing ICC estimates with 95% CI for nuclei and cell segmentation features that includes scalar counts of the cells by type. (**b**) Forest plot of ICC estimates with 95% CI for the cell proportion features. (**c**) ICC estimates for spatial features that includes pairwise neighborhood counts by cell types, coefficient of variation of Voronoi polygon area by cell type, mean and standard deviation of edge length of Delaunay triangulation by cell type, and Shannon diversity index by the cell type. (**d**) ICC values grouped by feature type (spatial features and segmentation). **e**) Bootstrapped mean ICC for all features grouped by comparisons C1 vs. C3, C1 vs. C2, and C2 vs. C3.

The cell-type specific features showed overall excellent agreement with a median [IQR] ICC estimate of 0.844 [0.770, 0.900]. The median ICC estimate of the cell proportion features was 0.876 [0.766, 0.906], and the median ICC estimates of the scalar counts of cells by the type was 0.839 [0.773, 0.895] (Fig. 2a,b). ICC estimates were lowest across the cross sections for the dead cells, and the non-neoplastic epithelial cells (Fig. 2a,b). The dead cells, non-neoplastic epithelial cells, and neutrophils were sparse cell types on a WSI, and it was observed that lower abundance correlated with lower ICC (Supplementary Fig. 3). ICC estimates were compared for cell type counts and proportions by their distance and the median [IQR] ICC estimates of cell counts and cell proportions for the cell types were 0.770 [0.742, 0.851] and 0.878 [0.833, 0.895] respectively between the distant cross sections (C1 vs. C3), compared to closer cross sections C1 vs. C2 (count = 0.818 [0.795, 0.878]; proportion = 0.906 [0.825, 0.905]), and C2 vs. C3 (count = 0.858 [0.803, 0.902]; proportion = 0.898 [0.895, 0.935]) (Fig. 2d).

The spatial features showed an overall excellent agreement with a median [IQR] ICC estimate of 0.873 [0.771, 0.933] (Fig. 2c). The pairwise neighbor count features showed a median [IQR] ICC estimate of 0.877 [0.785, 0.930]. ICC estimates were lower for the dead cells’ neighborhood count features (Fig. 2c). The median [IQR] ICC estimates for the pairwise neighborhood count features were the lowest for the distant cross sections C1 vs. C3 (0.852 [0.802, 0.903]), compared to closer cross sections C1 vs. C2 (0.893 [0.836, 0.944]) and C2 vs. C3 (0.920 [0.853, 0.937]) (Fig. 2d).

The coefficient of variation of Voronoi Polygon area for the cell types between the cross sections showed a median [IQR] ICC estimate of 0.885 [0.837, 0.929] with the distant cross sections C1 vs. C3 showing lowest ICC of 0.843 [0.790, 0.897] compared to closer sections C1 vs. C2 (0.914 [0.870, 0.950]) and C2 vs. C3 (0.880 [0.853, 0.918]) (Fig. 2d). The mean length of Delaunay triangulation edges for the cell types showed a median [IQR] ICC estimate of 0.894 [0.832, 0.951], with the distant cross sections C1 vs. C3 showing the lowest ICC of 0.845 [0.803, 0.906] compared to the closer cross sections C1 vs. C2 (0.924 [0.899, 0.952]) and C2 vs. C3 (0.879 [0.832, 0.937]) (Fig. 2d). The standard deviation of length of Delaunay triangulation edges showed a median [IQR] ICC estimate of 0.938 [0.905, 0.977], with distant cross sections C1 vs. C3 showing lowest ICC estimate of 0.927 [0.886, 0.951] compared to closer cross sections C1 vs. C2 (0.969 [0.940, 0.981]) and C2 vs. C3 (0.931 [0.909, 0.947]) (Fig. 2d). Finally, the Shannon diversity index showed an overall excellent agreement with median [IQR] ICC estimate of 0.878 [0.837, 0.922], with distant cross sections C1 vs. C3 showing the lowest median ICC [IQR] value of 0.844 [0.802, 0.880] compared to closer cross sections C1 vs. C2 (0.886 [0.869, 0.908]) and C2 vs. C3 (0.898 [0.862, 0.930]) (Fig. 2d).

All the nuclei, cell segmentation features, and spatial features were compared by the cross section distance using cluster bootstrapping. C1 vs. C3 (distant) compared to neighboring cross sections C1 vs. C2 and C2 vs. C3 showed the lowest mean ICC showing that the cross sections separated by the most distance resulted in the least reproducibility (Fig. 2d,e). Comparisons of mean differences of ICC estimates for all features by the distance C1 vs. C3, C1 vs. C2 and C2 vs. C3 using cluster bootstrapping showed significantly lower ICCs (p-value < 0.05) for the distant comparisons versus neighboring comparisons (Table 1). Cluster bootstrapping results at a feature level indicated that 92.5% of the features evaluated showed significantly lower ICCs (p-value < 0.05) for the distant comparisons C1 vs. C3 compared to the neighboring comparisons.

**Table 1:** Cluster bootstrapping results for distant and neighboring comparisons of feature ICCs.

Comparison	Bootstrapped Mean ICC value	Bootstrapped mean difference of ICC	Significance based on mean differences (*p*-values)
C1 vs. C3	0.7314	–	–
C1 vs. C2	0.7909	–	–
C2 vs. C3	0.8119	–	–
C1 vs. C3 vs. C1 vs. C2	–	0.0595	0.0308
C1 vs. C3 vs. C2 vs. C3	–	0.0805	0.0001
C1 vs. C2 vs. C2 vs. C3	–	0.0210	0.3898

### Reproducibility of feature embeddings derived from foundational models

**Fig. 3:**
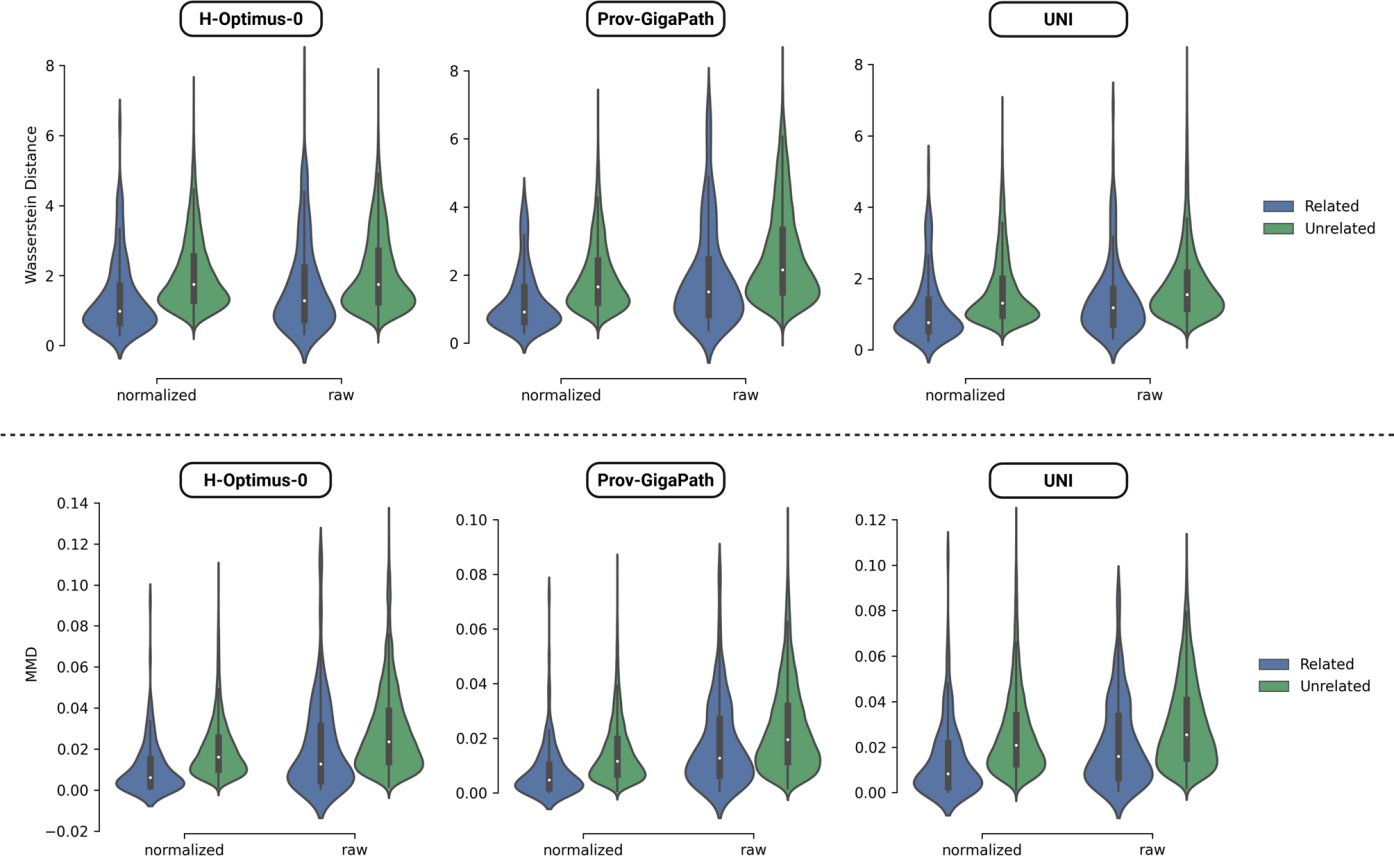
Comparison of feature embeddings from FMs across related slides from the samples and unrelated slides in our cohort for stain-normalized and raw slides.

The distribution of the distance metrics for the related and unrelated slides from FM embeddings is shown in Fig. 3. Across all the models, Wasserstein distance and the MMD values showed lower median values for the related samples compared to the unrelated samples (Fig. 3). For the H-Optimus-0 model, the median [IQR] Wasserstein distance between the sample related slides was 1.282 [0.739, 2.230] and the unrelated slides was 1.748 [1.248, 2.713]. The median [IQR] MMD for the H-optimus-0 between sample related samples was 0.013 [0.004, 0.031], and the unrelated sample pairs was 0.024 [0.014, 0.038]. For the Prov-GigaPath model, Wasserstein distance between sample related slides was 1.509 [0.835, 2.468], and unrelated slides was 2.159 [1.507, 3.330]. The MMD between sample related slides for the Prov-GigaPath model was 0.127 [0.006, 0.027], and the unrelated slides was 0.019 [0.011, 0.032]. Finally, for the UNI model, the Wasserstein distance between sample related slides was 1.177 [0.702, 1.700] and 1.551 [1.154, 2.166] for unrelated slides. The MMD between sample related slides was 0.016 [0.007, 0.034] and unrelated slides was 0.025 [0.015, 0.041].

For all the models evaluated in the study, the permutation test resulted in a p-value < 0.001 for the Wasserstein distance and the MMD metric, indicating that the related slides resulted in smaller values compared to the unrelated slides.

The sensitivity analysis to assess the changes in the FM embeddings for raw and stain normalized slides showed that the median MMD and median Wasserstein distance trended modestly lower for the normalized slides compared to the raw slides (Fig. 3). The permutation test for the stain normalized slides resulted in p-value < 0.001 between the related and unrelated slides, similar to the raw slides.

Evaluation of mean differences of Wasserstein distance and MMD estimates by the cross section pair-wise comparisons C1 vs. C3, C1 vs. C2 and C2 vs. C3 across the FMs using cluster bootstrapping showed significant differences for the distant comparisons (C1 vs. C3) vs. (C1 vs. C2) (Wasserstein-distance p-value: 0.0366, MMD p-value: 0.0342) and (C1 vs. C3) vs. (C2 vs. C3) (Wasserstein-distance p-value: 0.0144, MMD p-value: 0.0100), but not for the neighboring comparisons (C1 vs. C2) vs. (C2 vs. C3) (Wasserstein-distance p-value: 0.7402, MMD p-value: 0.6612).

## Discussion

Digital pathology offers vast potential to develop representation learning approaches for various tasks, including nuclei segmentation, malignancy classification, disease risk stratification, gene expression evaluation, mutation status prediction, and microsatellite instability assessment^39^. Slide preparation methods associated with digital pathology introduce several sources of variability, such as differences in staining, sectioning, institutional methods, and scanning platforms. There have been several published predictive DL models and FMs in the field of digital pathology with applicability to several medically relevant tasks, but there exists a lack of consensus about features and their reproducibility in sequential cross sections of a tissue sample. Similar feature reproducibility studies have emerged in the field of radiomics, where quantitative features have been evaluated in various conditions, including sequential sections, and specific reproducible and non-reproducible features have been highlighted^40^. However, there remains a lack of understanding of feature reproducibility in the field of digital pathology, especially when sequential cross sections are involved. Our study’s primary objective was to thoroughly evaluate feature reproducibility in digital pathology using sequential cross sections from a large set of tissue samples.

In this study, we characterized the reproducibility of a diverse set of features extracted from state-of-the art DL and FM models to capture both cellular and whole-slide level morphological information. With the features derived from nuclei segmentation as well as feature embeddings from FMs, we found a generally high level of agreement across the sequential cross sections. However, there are specific differences that warrant attention depending on the use case. For example, at the cell level, certain sparse cell types in our tissue samples, such as non-neoplastic epithelial cells, neutrophils, and dead cells, demonstrated lower reproducibility in cross section comparisons. Furthermore, with respect to spatial features, the sparsity and morphological variability of dead cells lead to considerable variation in the associated spatial features across sequential cross sections. Therefore, it is advisable to exercise caution when interpreting these specific sparse cell-related features due to their low reproducibility across sequential cross sections in 2D digital pathology. While most cellular-level and spatial features demonstrate high reproducibility, the comparisons revealed that the results are not identical. This suggests subtle intra-sample variability among all features across sequential cross sections. Depending on the specific application, these minor differences could be significant. When inferring cell-type proportions to evaluate the normalcy or malignancy of tissue, it is essential to recognize that variations in key markers, including cell type proportions, may occur depending on the specific cross section examined. These differences can be influenced by biological factors, leading to morphological discrepancies among tissue slices, or may be attributed to the sectioning process itself. Hence, we advise adopting careful approaches when determining thresholds for such classification tasks.

We also highlight that more distant cross sections expectedly demonstrate a higher variability compared to closer sections for the cell-level and spatial features. This is important to consider, as numerous studies have utilized digitized slides to analyze RNA gene expression and DNA mutation status^7,16–18,41–43^. Often, different tissue cross sections are frequently used for DNA and RNA extraction, as well as for slide digitization. The distance intervals between these sequential cross sections may lead to potential underestimation or overestimation of gene expression levels, as well as inaccuracies in predicting mutation status, especially when cellular and spatial features are integrated into model training. These variations may influence clinical decision-making processes. Digital pathology models have been used to estimate odds ratios, conduct survival analyses, and classify risk categories^1,2,4,6,10,11,13^, and the intra-sample variability in feature measurements due to cross sectional differences can potentially lead to attenuation of the odds ratios and may result in incorrect risk category assignments. To effectively address these impacts, we recommend that digital pathology studies consider multiple cross sections to aggregate estimates and facilitate robust conclusions Additionally, reporting measurement uncertainties alongside results and evaluating clinical endpoints through decision-curve analysis can provide robust strategies for managing potential measurement errors.

Finally, we also showed that the feature embeddings at the slide level from state-of-the art digital pathology FMs show reproducibility across the sequential cross sections. While the distance metrics were significantly lower for all related compared to the unrelated samples, it is notable that the distributions showed a heavy overlap. This may be attributable to three primary reasons: first, tissues have complex 3D structures. In some instances, a sequential section might miss or capture different microenvironments and there could be sectioning related artifacts. As an example, we highlight the sequential cross sections from same tissue for one of the samples in our cohort and it can be seen that the sequential cross sections are morphologically varied (Supplementary Fig. 4). These morphological differences will result in dissimilar feature embeddings, and hence might inflate the distance metrics calculated for the related samples in our study, resulting in overlapping values with unrelated samples. Secondly, there could be staining differences between sequential cross sections that potentially affect the embeddings retrieved, resulting in related samples yielding distance metrics that show dissimilarity. To confirm if this was a reason, a sensitivity analysis was performed by using stain normalized slides and the results and conclusions remained unchanged (Fig. 3). Notably, H-Optimus-0 model results pre- and post-normalization were similar (Fig. 3), indicating that this model is robust to staining differences compared to other models. Finally, the foundation models retrieve embeddings and often these embeddings are inputs towards downstream classification or regression models. Given that our study involved a collection of normal prostate tissue samples, it is possible that the high level of similarity in embeddings from the FMs across samples reflects this degree of homogeneity in sample characteristics. This final hypothesis requires further validation with other tissue types and biological conditions.

With the morphology and the topology changing between different cross sections due to the 3D nature of human tissues^14^, it is not unexpected that inferences might differ based on the cross section being examined in a study. Digital pathology is accessible in both 3D and 2D formats, facilitating detailed quantitative analyses tailored to specific medically relevant inquiries^44^. 3D pathology preserves the natural dimensions of the human tissue and has been demonstrated to show an improved performance over 2D pathology for patient prognostic and predictive assessments^14^. However, clinical adoption of the 3D digital pathology has several challenges, including the need for extensive validation, regulatory approval, and availability of diverse datasets for model training and evaluation^14,44^. Several 3D reconstruction algorithms have been developed to leverage sequential 2D cross sections to compose a 3D tissue, with potential applications in several medically relevant contexts; however, the method-specific parameterization and the diversity of tissue types affect generalizability^45,46^. In contrast, 2D digital pathology has been widely adopted and several imaging platforms have been developed and been approved for medical use^2–4,10,12,13,15,21,47^. 2D digital pathology involves slicing an inherently 3D human tissue to create a cross section and placing it on a glass slide, staining it, and digitizing it using scanners to create WSIs^47^. Recently several 2D digital pathology and associated computational models have been demonstrated to have clinical grade performance for various types of tasks^1,48^, but the variability due to the morphometric features across cross sections and its potential to affect the model performance is not well characterized.

Several study limitations warrant mention. First, we were unable to compare the pixel-by-pixel variability across the sequential cross sections. Due to the inherently destructive nature of 2D pathology, the tissue size and morphology changes across the sequential cross sections. When the sequential cross sections are digitized to yield WSIs, it is possible that the dimensions of these sequential cross sections do not match. Image registration techniques could improve WSI alignment and reduce this technical source of variation, particularly for FM feature extraction. Another technical limitation in our study is that the study cohort is from a single institutional center. It has been previously established that FMs, including the ones used in our study, might have generalizability limitations due to medical center differences^49^. To standardize reproducibility studies, it is essential that future research incorporates data from multiple medical centers. We believe our methods for evaluating feature reproducibility in digital pathology provide a valuable framework for similar studies.

Additionally, although the ostensibly similar sections show an overall high agreement in features, the subtle variations observed may be inherently biological, leading to morphological changes in one tissue section that are not present in other sequential sections. We recommend that users of digital pathology and associated image representation learning take these sample-level biological and population-level methodological differences into account when drawing conclusions. Our analyses were restricted to benign prostate tissue, and our findings may not generalize to other tissue types or to malignant tissue contexts. Finally, we were only able to examine the influence of inter-slide physical distances defined by our sectioning protocol (approximately 50 and 100 μm), and our estimates of reproducibility are likely conservative relative to true serial slices. Future studies that examine larger numbers of true serial slices would be able to provide more granular assessments of intra-sample feature variability as a function of inter-slide distances.

## Conclusion

In conclusion, our study establishes high reproducibility of features extracted from DL and FM methods across sequential cross sections, while crucially highlighting higher intra-sample variability of certain features with distant sections. We also underscore the subtle differences of cell-level and spatial features from sequential cross sections and suggest key considerations in inferences of these features and usage in downstream classification and regression models. Finally, we anticipate that our results will stimulate development of approaches that account for distances across the sequential cross sections in adjusting the extracted features, thereby resulting in robust feature inferences.

## Supplementary Information

Below is the link to the electronic supplementary material.

Supplementary Material 1

## Data Availability

Raw slides and data will be made available upon reasonable request. The code used in the study has been made available in GitHub: https://github.com/jagadhesh89/Feature_Reproducibility_Digital_Pathology.
