# Supplementary material for: Reproducibility of digital pathology features extracted from deep learning and foundational AI models on sequential tissue slides
Source: Sci Rep. 2025 Dec 6;16:1429. doi: 10.1038/s41598-025-30947-w (PMC12796287; doi:10.1038/s41598-025-30947-w)
Supplement: Supplementary file 1 — Supplementary Material 1 [file 41598_2025_30947_MOESM1_ESM.docx]

# Supplementary Materials:


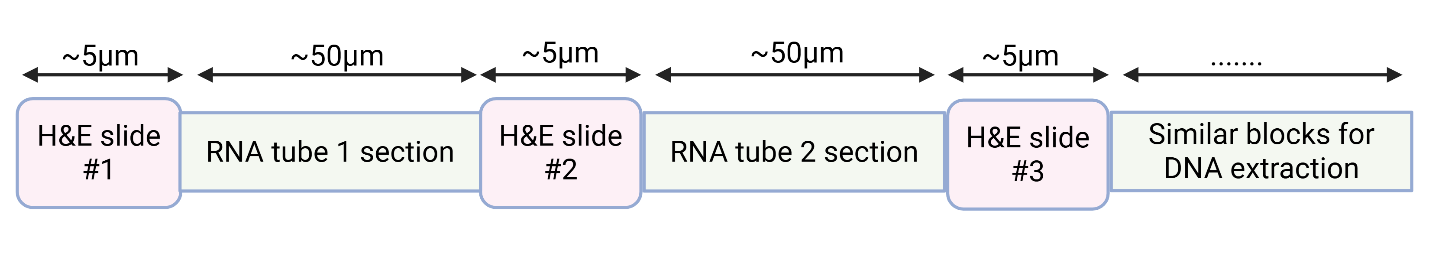


**Supplementary Figure 1**: Sequential cross sections prepared for the study. The first three slides prepared in the sectioning protocol were chosen to investigate reproducibility of digital pathology features.


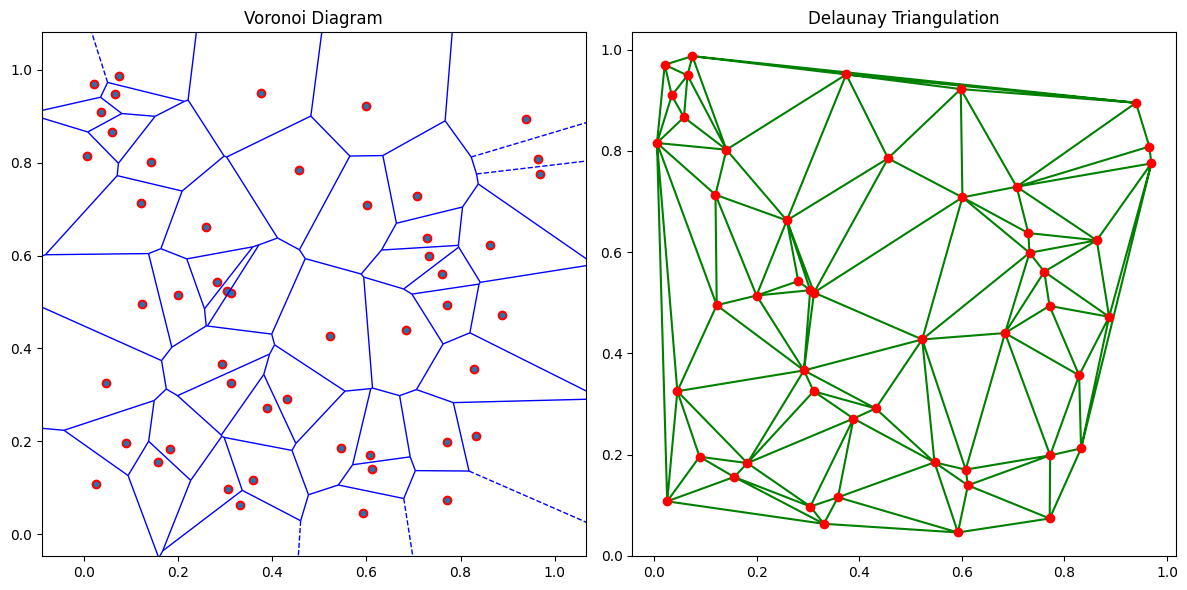


**Supplementary Figure 2**: 50 random centroids were generated and the Voronoi polygon area and the Delaunay triangulation are demonstrated on the centroids. It can be observed that the outer centroids have a wider area in the Voronoi polygons compared to the inner centroids.


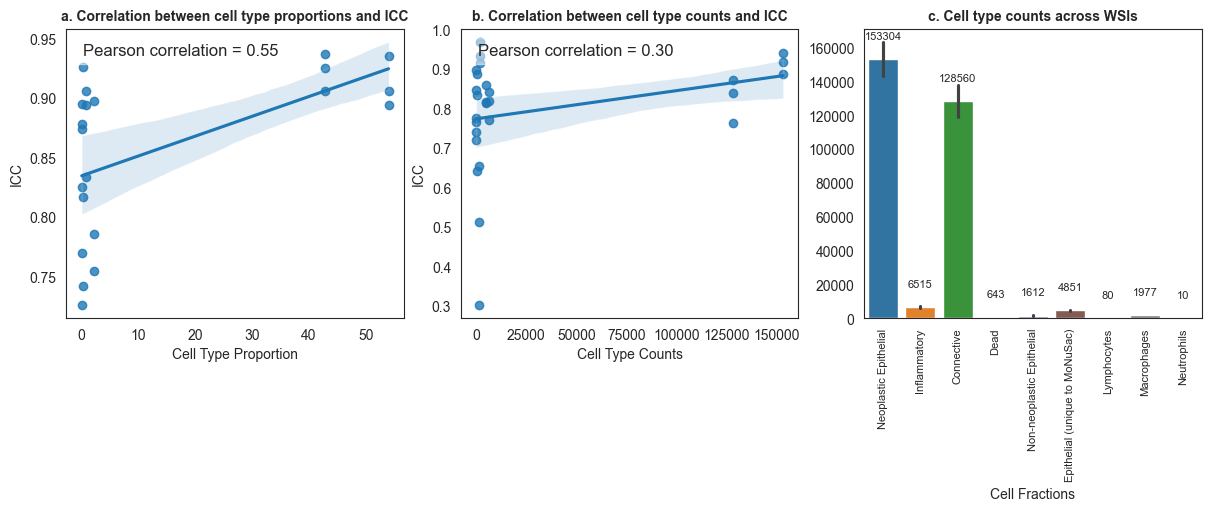


**Supplementary Figure 3:** **a.** Correlation between cell type proportions and ICC in a randomized third of the cohort (16 patients, 3 WSIs each, total 48 WSIs). **b**. Correlation between cell type counts and ICC in a randomized third of the cohort (16 patients, 3 WSIs each, total 48 WSIs). **c**. Cell type counts across WSIs in a randomized third of the cohort (16 patients, 3 WSIs each, total 48 WSIs).


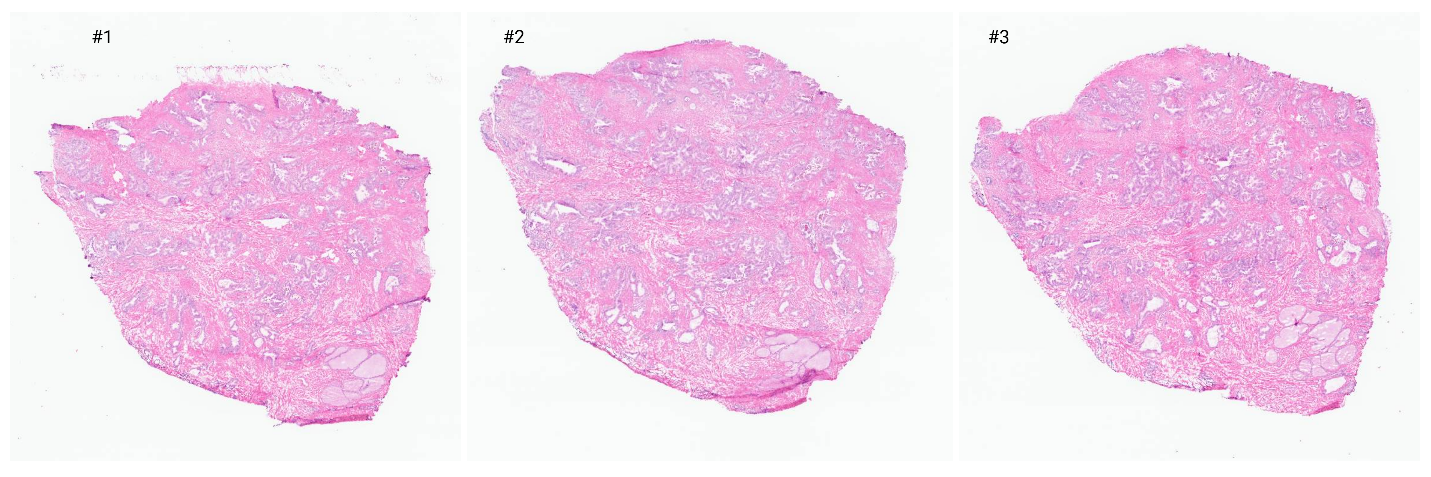


**Supplementary Figure 4**: Sequential cross sections of same tissue look morphologically different in many regions.

**Supplementary Table 1**: Features extracted from the digitized sequential cross sections

| **Feature category** | **Feature Name** | **Cell Types (if applicable)** | **Total number of features** |
| --- | --- | --- | --- |
| **Cell and nuclei level** | Cell type Counts | 1. Neoplastic Epithelial (from PanNuke) 2. Non-Neoplastic Epithelial (from PanNuke) 3. Neutrophils 4. Lymphocytes 5. Other inflammatory cells 6. Connective cells 7. Epithelial cells (unique to MoNuSac) 8. Dead Cells | 8 |
|  | Cell type proportions | Same as above | 8 |
| **Spatial Features** |  |  |  |
|  | **Category 1 (Size of nuclei by cell type):** Coefficient of Variation of Voronoi Polygon area by each cell type | 1. Epithelial cells 2. Connective cells 3. Inflammatory cells 4. Dead cells 5. Macrophages | 5 |
|  | **Category 1 (Size of nuclei by cell type):** Mean edge length of Delaunay triangulation of nuclei by each cell type | Same as above | 5 |
|  | **Category 1 (Size of nuclei by cell type):** Standard deviation of Delaunay triangulation of nuclei by each cell type | Same as above | 5 |
|  | **Category 2 (Neighborhood counts):** Pairwise neighborhood counts (to quantify other neighboring cell type to each cell type) | Same as above | 25 (5 X 5) |
|  | **Category 3 (Neighbor diversity by cell type):** Shannon diversity index (to assess abundance or lack of diversity of cell types for each cell type) | Same as above | 5 |
| **Feature embeddings from foundational models** | Feature vector of $m \times1024$ dimensions, where $m$ = number of $224 \times224$ tiles in the WSI |  | $n \times m \times1024$ , where $n=3$ (number of FMs: UNI, Prov-GigaPath and H-optimus-0) and $m$ = number of $224 \times224$ tiles in the WSI |

**Supplementary Table 2**: Cell type merging rules for assessing feature reproducibility.

| **Original cell type** | **Larger cell type** | **Merged into cell type** |
| --- | --- | --- |
| Neutrophils | Inflammatory | Inflammatory |
| Lymphocytes | Inflammatory | Inflammatory |
| Neoplastic epithelial (from PanNuke) | Epithelial | Epithelial |
| Non-neoplastic epithelial (from PanNuke) | Epithelial | Epithelial |
| Epithelial unique to MoNuSac | Epithelial | Epithelial |

Supplementary Text

Standard Operating procedure for H&E slide staining

- Rehydration step
  - Place slides in absolute alcohol for 30-60 seconds
  - Transfer slides to a 2^nd^ absolute alcohol for 30-60 seconds
  - Transfer slides to 95% alcohol for 30-60 seconds
  - Transfer slides to 70% alcohol for 30-60 seconds
  - Rinse slides in tap water for at least 30 seconds
- Hematoxylin stain
  - Place slides in Hematoxylin stain for 5 minutes
  - Rinse slides in tap water for at least 3-5 minutes, rinsing until tap water runs clear
  - Place slides in ammonia water for 30 seconds
  - Rinse slides in tap water for 30 seconds to 2 minutes
- Eosin stain
  - Place slides in 70% alcohol for 30-60 seconds
  - Transfer slides to 95% alcohol for 30-60 seconds
  - Transfer slides to Eson stain for 30-90 seconds
  - Tilt rack above Eosin tray for a few seconds to drain excess stain prior to moving to dehydration steps
- Dehydration
  - Place slides in 95% alcohol for 30-60 seconds
  - Transfer slides to second 95% alcohol for 30-60 seconds
  - Transfer slides to third 95% alcohol for 30-60 seconds
  - Transfer slides to absolute alcohol for 30-60 seconds
  - Transfer slides to second absolute alcohol for 30-60 seconds
  - Place slides in Xylene for at least 5 minutes
  - Transfer slides to second Xylene for at least 5 minutes
  - Transfer slides to third Xylene for at least 5 minutes
  - Slides should be manually coverslipped if a sticker label is lifting off of the slide, sticker labeled slides are left in Xylene longer than 15 minutes for the washes, or if a sticker label is handing over the edge of the slide
